# Comparative study of several sinusitis experimental modelling techniques in rabbits

**DOI:** 10.1016/S1808-8694(15)30122-1

**Published:** 2015-10-19

**Authors:** Henrique Olival Costa, Giulliano Enrico Ruschi e Luchi, Arthur Guilerme Augusto, Marilia Castro, Flavia Coelho de Souza

**Affiliations:** a Otorhinolaryngologist and Head & Neck Surgeon. Doctor in Otorhinolaryngology. Adjunct Professor in thede Otorhinolaryngology Department of the São Paulo Santa Casa. Coordinator of the Graduate Program in Otorhinolaryngology at the São Paulo Santa Casa; b Otorhinolaryngologist, Master’s degree student in ORL, Sao Paulo Santa Casa. Physician; c Associate Professor, Professor in the Otorhinolaryngology Graduate Program, Sao Paulo Santa Casa; d Pathologist, Sao Paulo Santa Casa; e Veterinary Doctor, Master’s degree in Health Sciences, UNIFESP. Medical Science School, Sao Paulo Santa Casa

**Keywords:** rabbits, experimental, model, rhinosinusitis, sinusitis

## Abstract

Experimental models for clinical studies of rhinosinusitis are needed. **Aim:** to define a reliable, solid and reproducible experimental model for inflammatory rhinosinusitis with no innoculation of infectious agents in rabbits. Study design: Experimental. **Material and Method:** Twenty 20 rabbits were divided into 4 groups submitted to 4 different interventions: the placement of a unilateral nasal fossa sponge, unilateral obliteration of the nasal ostium with cyanoacrylate, unilateral placement of antigens in the maxillary cavity and unilateral placement of blood in the maxillary cavity. The animals were monitored for 15 days and then anesthetized and sacrificed; the maxillary sinuses were evaluated histologicaly and results were compared with controls and between the intervention groups. **Conclusion:** Sponje and glue as agents of meatal obstruction and toxoid aplied in the antrum are efficient as methods for rhinosinusitis modeling. Blood was not efficient in producing sinusitis.

## INTRODUCTION

The prevalence of airway infectious-inflammatory diseases is extremely high in any population group (about 25% of a given population has allergic rhinitis and each inhabitant has on average four colds per year; there are 680,000 episodes of acute rhinosinusitis per year in Brazil) and the cost of medication is high (the average cost of treating acute rhinosinusitis is R$ 20.00). In the USA it is estimated that the annual expense with this disease is about 5 billion dollars.^1^, ^2^

There are various options for treating rhinosinusitis, starting with topical and systemic anti-inflammatory drugs, astringent medication, mucolytic agents, antiseptic substances and antibiotics.

Added to the natural difficulty of classifying regional pathological processes, which frequently results in grouping together infections, allergies and anatomical alterations, pathogenic factors may coexist in the same clinical case. This results in various misconceptions in the clinical study of rhinosinusitis.

Given this situation, we believe that new experimental models are needed for adequate clinical studies of rhinosinusitis.

The literature contains various attempts to create animal models for this type of study, rodents being the most common. Apparently there is a pathophysiological reason for this, as the nasal anatomy and physiology of these animals is similar to that of human beings.

Rodents that have been studies include rabbits, albino guinea-pigs, and Wistar and Sprague-Dawley mice; the former are preferred in studies involving surgery or other invasive procedures - although mortality is high due to stress - and the latter are considered superior for middle and long-term studies, given their resistance. In these terms guinea-pigs are somewhere in the middle between rabbits and mice.

There are a variety of procedures to cause paranasal sinus inflammation similar to common rhinosinusitis. Measures have included obliteration of nasal fossae, occlusion of sinus drainage ostia, instillation of inflammation mediators in sinuses and placing materials that become culture media in the nasal fossae. Aside from these aseptic methods, other studies have used in vivo inoculation of infectious agents (fungi, bacteria and viruses.^1^, ^2^

There are, however, no comparative studies in the literature where the method and the morphological anatomical pathological description is made in detail. Furthermore, there is an excessive variety of procedures to obtain inflammation.

The fact that many forms of reaching a final result have been described, allied to the paucity of methodological clarification and comparison between such methods, has led us to conclude that we lack sufficient information to safely design animal experiments that require modeling of rhinosinusitis, which increases the possibility of performance bias and control difficulties.

Our aim in this study was to establish a reliable, reproducible and consistent experimental model for inflammatory rhinosinusitis in rabbits without inoculating infectious agents.

## MATERIAL AND METHOD

Our study was done in an animal room on experimental animals, under veterinary supervision, after approval by the Research Ethics Committee for Animal Experiments of the Sao Paulo Santa Casa (protocol number 117).

Animals chosen for this study were New Zealand rabbits aged 6 months and weighing between 2.3 and 3.5 kg; the nasal anatomical structure of these animals is similar to that of human beings, and offer favorable manipulating, monitoring and evaluating conditions.

Twenty rabbits were placed in four groups of five animals each. Each group underwent a different procedure to obtain a controlled inflammatory response in the paranasal sinuses.

The animals were weighed before the procedure and daily until being sacrificed. Daily food intake was monitored and auricular temperature was measured twice daily.

Parameters indicative of the clinical condition were used to assess the animals, as follows: food acceptance, weight and temperature.

Animals were monitored during 15 days following the initial procedure and were then anesthetized and sacrificed.

### Procedures

Animals were anesthetized with intramuscular Zoletil® at 0.4 ml/Kg before the surgical procedure and kept on spontaneous ventilation.

A nasal speculum was placed to expose the nasal cavity of the side chosen randomly immediately before the procedure.

After placing the speculum in groups 1 and 2, 0.8ml of xylocaine and epinephrin 2%® was sprayed in the nasal fossa that was to be manipulated and left for 3 minutes before starting the procedure.

The animals were subjected to the following procedures, according to the intervention group:

**Group 1:** Composed of 5 animals. In this group a bath sponge was cut to measure 4 cm x 0.5 cm x 1 cm and inserted in one of the nasal fossae, chosen randomly. The procedure was considered as ended when we verified that the sponge had been placed correctly and that it filled all of the nasal fossa.

**Group 2:** Composed of 5 animals. In this group cyanoacrylate was placed in the infundibulum of a randomly chosen nasal fossa to occlude the nasosinusal ostium. The procedure was considered as ended when total occlusion of the ostium was established.

**Group 3:** Composed of 5 animals. In this group 1 ml of peripheral venous blood was taken and instilled by percutaneous puncture into one of the maxillary sinuses chosen randomly. The procedure was considered as ended when blood was fully instilled in the paranasal sinus.

**Group 4:** Composed of 5 animals. In this group 1 ml of laboratory-prepared toxoid (containing 50% of streptococcal and 50% of staphylococcal toxoid) was instilled by percutaneous puncture in a randomly chosen maxillary sinus. The procedure was considered as ended when the antigens were fully instilled in the paranasal sinus.

In all of the interventions the maxillary sinus and the nasal fossa that did not undergo the procedure were used as controls.

Fifteen days later the animals were anesthetized and for euthanasia.

At this point the facial middle structure was removed en bloc and placed in formaldehyde for pathology.

### Pathology examination

The facial middle structure blocs were frozen and cut in coronal serial sections 3 mm from the anterior wall of the maxillary sinus to its posterior wall; these sections were studied histologically.

Slides were prepared by one pathologist and assessed by three qualified professionals who were blinded to the animal groups of the study.

Qualitative and quantitative defining parameters for inflammation were the presence of neutrophil and eosinophil clusters, regions with loss of lining cells, caliceal cells and nitric oxide synthetase (iNOS)-positive induced inflammatory cells.

## RESULTS

All of the animals in this study developed unilateral yellowish rhinorrhea by the 15th day after the procedure.

Most of the animals presented mild weight loss in the first day after the procedure, progressively regaining normal weight throughout the study period.

Temperature varied not more than 0.8ºC throughout the study period; no animal presented fever.

**Figure 1:**
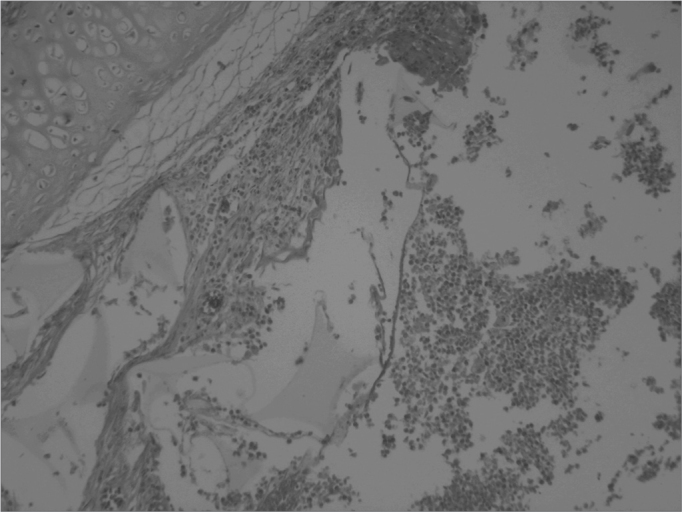
Maxillary sinus mucosa showing a moderate to intense infectious inflammatory process and a foreign body (sponge). HE stain, 40X.

**Figure 2:**
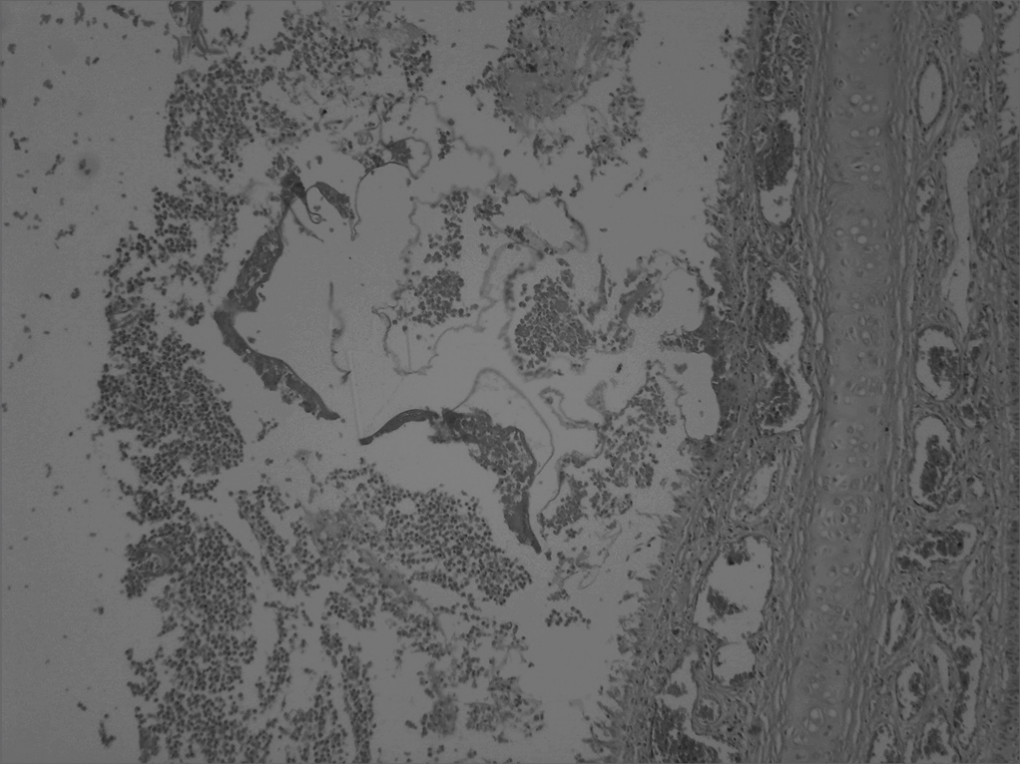
Cross-section of the rabbit maxillary sinus mucosa showing intense inflammation, surrounded by accumulated fibrin and red blood cells; the amorphous material was glue. HE stain, 40X.

Pathology of the facial middle structure reveled an acute intense inflammatory process of the nasal cavity and maxillary sinus in group 1 that surrounded a fragmented yellowish foreign body (the sponge), with an ulcerated epithelium and capillary neoformation in the chorion that characterizes granulation tissue (Figure 1). In group 2 animals pathology revealed a moderate acute inflammatory process, with macrophages that permeated the nasal cavity and the maxillary sinus and surrounded a light pink filamentous foreign body (cyanoacrylate), with epithelial ulceration, capillary neoformation and a lymphoplasmocytic infiltrate in the chorion (Figure 2). In group 3, three animals presented a mild acute purulent inflammatory process and some red blood cells in the nasal cavity and maxillary sinus. In the other two animals only blood cells were found in the nasal cavity and the maxillary sinus lumen, with no report of inflammation. In group 4, pathology reported a mild purulent acute inflammatory process and red blood cells within the nasal cavity and the maxillary sinus lumen. The contralateral side was used as a control in all of the animals; no inflammation was described in these specimens.

## DISCUSSION

Various papers about the most frequent changes in the nasosinusal mucosa as a result of inflammation and the main aspects of therapy are available in the literature. There are also ample debates in these studies about which research model should be used, taking into account which animal should be used and the means of provoking rhinosinusitis. We have made some studies in this area in an attempt to standardize an experimental model for rhinosinusitis, presenting the methodology in detail, to eliminate bias in such studies.

Authors that have investigated this area have used varied means of designing their models. Some have introduced pathogenic bacteria within facial sinuses, with or with no drainage ostium blockage. Even though efficient, this approach has operational difficulties, requiring a protected environment to manipulate pathogenic species; its use is, therefore, unfeasible in less well-equipped research facilities.

Cable (2000)^3^ introduced Pseudomonas strains surgically in rabbit paranasal sinuses in a study on the efficacy of corticosteroids in the treatment of rhinosinusitis. Stierna (1991)^4^ compared pathological changes in the sinusal mucosa of rabbits inoculated with secretions containing Streptococcus pneumoniae and Bacteroides fragilis, after maxillary ostium blockage. The author noted more intense and pronounced changes in the mucosa that had received Bacteroides secretions. In these cases there was periosteal thinning, bone neoformation, more pronounced rhinorrhea and increased lactate levels (suggesting anaerobic metabolism), besides the expected changes resulting from inflammation. Westrin (1992)^5^ induced sinusitis in rabbits by blocking the sinus ostium and inoculating the sinus with a solution containing Bacteroides fragilis. A further conclusion of this paper was that inflammatory damage was increased compared to pneumococcal infection.

Maeyama (1981)^6^ used a Staphylococcus solution instilled percutaneously. There was a high incidence of sinusitis two weeks later, demonstrated by electron microscopy and histology. Johansson (1988)^7^ used a rabbit model similar to that used in the current study, obstructing the ostium and instilling 1 ml of Streptococcus toxoid within the sinus. Sinusitis developed in all of the animals. In two other groups the ostium was blocked singly or toxoid was instilled within the sinus without blocking the ostium; in these cases infection did not develop. These results are different from our findings, as we found a high rate of rhinosinusitis in toxoid-inoculated rabbits with open ostia.

Kara (2002)^8^ used rabbits and produced sinusitis only in animals in which the ostium was blocked by an absorbable hemostatic sponge containing a Staphylococcus solution. In the groups where the ostium was obstructed, but the sponge was sterile, no disease developed until the twentieth week of observation. In 2003, this author published a review of the methodology for developing a sinusitis model in rabbits, suggesting that this would be a reliable model for human sinusitis. This study also did not attain our results, given that mere nasal fossa obstruction with a sponge was enough to produce the model.^9^

Marks (1997)^10^ attempted to standardize a model of bacterial sinusitis in rabbits by introducing a foreign body in the nasal cavity followed by instillation of a solution containing pathogenic bacteria. Sinusitis developed in 50% of the animals, where the peak inflammatory reaction was seen in the second week, after which it regressed progressively until the fourth week, when chronic pathological changes in the mucosa were observed. We should repeat this type of longer lasting study, as the authors were unable to maintain inflammation for long; such a model would not be adequate for longer term studies. Drettner (1987)^11^ developed a sinusitis model in rabbits by surgically exposing the maxillary sinus, blocking the nasal ostium and introducing Streptococcus pneumoniae colonies within the sinus. After four days the colonies were removed and the maxillary sinuses were assessed histologically. There was thinning of the mucosa, dilatation of venules, increased blood flow and granulocyte infiltration. No bacteria were found in the mucosa.

Dufor (2005)^12^ attempted to standardize a fungal sinusitis model in rabbits, and concluded that this condition may be induced by blocking the ostium and inoculating the sinus with fungal strains. In the group where the ostium was not blocked, pathology did not develop notwithstanding fungal inoculation.

Cetin (2002)^13^ demonstrated that rhinosinusitis developed in rabbits following catheterization of the nasal fossa. The animals were evaluated at the end of the first, second and fourth weeks by computed tomography imaging, microbiology and histopathology. The author concluded that nasal fossa catheterization is a predisposing factor for rhinosinusitis, in proportion to the duration of probe permanence and its width.

Hinni (1992)^14^ assessed early - up to the fifth day - changes in function of the sinusal mucosa resulting from ostium blockage and the inoculation of bacterial strains commonly involved in these inflammatory processes. The author found a decrease in ciliated cells and an associated change in mucocilliary activity, depending on the microorganism, which was an important factor in predisposing the development of acute sinusitis.

Hurley (2001)^15^ conducted a quantitative analysis of leukotrienes, indicating failures in the sinusitis model in rabbits that had been used widely since the 80s. This author stated that the model does not reproduce reliably human sinusitis, given that the bacteria commonly involved in human disease are not as pathogenic as those in rabbit sinusitis. This author also stated that the maxillary sinus in the model is too small, which limits the material available for study. This opinion contests Kara’s^9^ published conclusions.

All of these studies show that there are various forms of creating an experimental model for rhinosinusitis, with the caveat that there are many variables involved. We considered the possibility of establishing an inexpensive and easily conducted experimental model for rhinosinusitis, using easily obtained material, which would be essential for experimental in our context.

We were able to attain results that are similar to those found in the literature, using procedures that are adequate for our context, and attaining sufficient histological consistency.

The use of a sponge and toxoid appears to have the best logical basis and reliability, and we intend to increase the duration of our investigation of this method.

## CONCLUSION

We concluded that a rhinosinusitis model in rabbits observed for 15 days can be standardized. Among the four methods we used, a more intense inflammation was obtained by introducing a sponge into the nasal fossa; on the other hand, blood placed in the nasal cavity resulted in little inflammation.
